# A Two-Year Study on Swifts (*Apus* spp.) as Bioindicators of Environmental Antimicrobial Resistance Within a One Health Framework

**DOI:** 10.3390/pathogens15010097

**Published:** 2026-01-16

**Authors:** Erika Esposito, Raffaele Scarpellini, Tiziano De Lorentis, Anna Zaghini, Giovanna Marliani, Elisabetta Mondo, Stefano Pesaro, Silvia Piva

**Affiliations:** 1 Department of Veterinary Medical Sciences, Alma Mater Studiorum-University of Bologna, Via Tolara di Sopra, 50, Ozzano dell’Emilia, 40064 Bologna, Italy; erika.esposito6@unibo.it (E.E.); tiziano.delorentis@studio.unibo.it (T.D.L.); anna.zaghini@unibo.it (A.Z.); elisabetta.mondo2@unibo.it (E.M.); silvia.piva@unibo.it (S.P.); 2 Department of General Psychology, University of Padova, Via Venezia, 8, 35131 Padova, Italy; giovanna.marliani@unipd.it; 3 Department of Agricultural, Food, Environmental and Animal Sciences, University of Udine, Via Sondrio, 2a, 33100 Udine, Italy; stefano.pesaro@uniud.it

**Keywords:** antimicrobial resistance, wildlife, swifts, wild birds, migratory birds, AMR, resistant bacteria, Bacillales, Enterobacterales, Africa, environment, surveillance

## Abstract

Antimicrobial resistance (AMR) is a global threat to human, animal and environmental health, underscoring the need for integrated surveillance to understand its dynamics and ecosystem interactions. This study investigated the potential of swifts (*Apus* spp.), long-distance migratory birds, as valuable bioindicators of environmental AMR dissemination. Four sampling sessions were conducted over two years (2023–2024) at a wildlife rehabilitation center in Trieste, Italy. Buccal and cloacal swabs were collected from 47 swifts: 10 sampled at arrival and 37 before autumn migration. Swabs were streaked on selective media for targeted isolation of Enterobacterales, Bacillales and Lactobacillales, yielding 168 bacterial isolates. Bacteria were identified using MALDI-TOF and antimicrobial susceptibility was assessed through disk diffusion method, using ECOFFs values or “no inhibition zone” criterion. Of the 168 bacterial isolates, 51 (30.36%) were non-wild type (NWT), with highest percentages of NWT isolates for clarithromycin (33.33%), erythromycin (31.50%), clindamycin (21.88%) and tetracycline (14.29%). Methicillin-resistant staphylococci (45.83%) and carbapenem NWT isolates (9.38%) were also detected. Bacillales isolates showed significantly higher NWT proportion (58.33%; *p* < 0.0001) compared to Enterobacterales and Lactobacillales. These findings, in clinically healthy non-antimicrobial treated swifts, suggest environmental exposure to resistant bacteria, and support a possible role of swifts as bioindicators of environmental AMR contamination, highlighting the need to strengthen environmental AMR surveillance within a One Health perspective.

## 1. Introduction

Antimicrobial resistance (AMR) constitutes a natural adaptive mechanism that allows microorganisms, including bacteria, fungi, parasites and viruses, to survive in the presence of antimicrobial agents [1]. Nowadays, AMR represents a critical global threat to both human, animal and environmental health. It is estimated that by 2050, AMR could potentially become the primary cause of death worldwide, since the number of deaths directly linked to AMR has risen to more than 1.2 million in 2019 and are forecast to increase to approximately 10 million per year by 2050 [2]. Major drivers of AMR include the overuse or misuse of antimicrobials, mainly in human and veterinary medicine [3]. Moreover, external pressures such as intensification of industrial activities and rapid urbanization can create selective pressures in the environment and facilitate the spread of resistant strains, contributing to the dissemination of resistance genes in bacterial populations and ecosystems [4]. In response to AMR, mitigation strategies recognizing the interdependence of human, animal and environmental health are needed, as highlighted by the global “One Health” approach [5]. Environmental monitoring of AMR has been recognized as crucial to understanding the resistance diffusion mechanisms, the factors that influence the phenomenon and the interactions between all the compartments of the ecosystem [6]. Numerous studies have emphasized the importance of AMR surveillance also in wildlife, as wild animals are not conventionally exposed to antimicrobials, but the presence of resistant bacterial strains in their microbiota could be closely related to the presence of AMR in their surrounding ecosystems. Wild animals are increasingly studied, considered bioindicators, sentinels, reservoirs and vectors of AMR at the human–animal–environment interface. Wild migratory birds are of particular interest in this context, since they could exhibit long-range movements, potentially acting as silent vectors between different geographical regions, and easily being exposed to resistant bacteria and antimicrobial resistance genes (ARGs), making them possible good bioindicators able to reflect the presence of an AMR environmental contamination [7]. Certain bacterial orders, including Enterobacterales, Bacillales and Lactobacillales, are commonly part of the wildlife gut microbiota. However, some members of these groups can act as opportunistic or pathogenic bacteria, occasionally causing infections [8,9,10]. Therefore, investigating their antimicrobial resistance profiles in wild birds provides insights into both environmental AMR and potential risks of bacterial infections in wildlife. Among migratory species, the swift (order Apodiformes, family Apodidae, genus *Apus*) is considered a long-distance migratory bird, moving from sub-Saharan Africa during wintering period over to Europe for breeding [11]. Specifically, in Europe, its arrival is in early spring, spending the breeding period in colonies in urban and peri-urban areas and raising their young before departing in late summer [11,12]. Despite their unique potential resulting from their migration routes and ecological behaviors to the best of our knowledge, no studies have yet investigated their role as bioindicators of the spread of AMR. Hence, this research aimed at two main objectives: (i) to investigate the antimicrobial resistance patterns of selected commensal bacterial orders (Enterobacterales, Bacillales and Lactobacillales) isolated from swifts; and (ii) to assess the potential role of swifts as bioindicators of environmental AMR.

## 2. Materials and Methods

### 2.1. Population Sampling and Data Collection

A total of four sampling sessions were conducted over two consecutive years (2023–2024) during two distinct moments corresponding to the swift’s migratory cycle: (i) at the arrival of swifts in Italy (July 2023; June 2024), and (ii) in late summer (September 2023; August 2024), after the breeding season and before autumn migration. According to the sampling period, swifts were classified in two groups to evaluate possible differences in AMR carriage before and after the breeding season. Moreover, age distinction was also assigned: adult swifts at the arrival in Italy, adult swifts after the breeding season, and juvenile swifts born in Italy. Samples were collected at the wildlife rehabilitation center “Liberi di Volare” in Trieste (Friuli-Venezia Giulia, Italy), where the swifts were brought for their recovery and sampled at least few days after. Only clinically healthy birds that did not receive any antibiotic treatment in the previous 90 days were included in the study. For each bird, a buccal and a cloacal swab was collected using Copan ESwab^®^ with Amies liquid transport medium (Copan Italia S.p.A., Brescia, Italy). Detailed signalment data for each individual were recorded, such as bird’s identifier name, species classification (common swift/pallid swift/alpine swift), age (adult, nestling or fledgling), sex (male/female), as well as sampling date. After sampling, the individuals were released back into the wild; therefore, it was not possible to re-sample the same birds at both sampling periods. The swabs were sent under refrigeration conditions within 24 h to the Veterinary Bacteriology Laboratory (VeLaBac) of the Department of Veterinary Medical Sciences (University of Bologna) and processed within 24–48 h.

### 2.2. Bacterial Isolation and Identification

From cloacal swabs, ten microliters (10 μL) of the Amies liquid transport medium were streaked onto MacConkey agar (Oxoid, Basington, UK) for selective isolation of Enterobacterales, while 10 μL from oral swabs were streaked onto Mannitol Salt Agar (Oxoid, Basington, UK) for selective growth of Bacillales and Lactobacillales. All plates were incubated aerobically at 40 ± 1 °C overnight, a temperature selected to approximate the birds’ physiological body temperature and to favor the growth of their commensal bacteria. Following 24–48 h of incubation, the colonies isolated on the different agar plates were evaluated based on their morphology and lactose/mannitol fermentation, cloned on Tryptone Soy Agar (Oxoid, Basington, UK) and incubated aerobically at 37 ± 1 °C overnight. After 24 h, species level identification of each isolate was assessed using the matrix-assisted laser desorption–ionization time-of-flight mass spectrometry method (MALDI-TOF MS) (Biotyper, Bruker Daltonics, Billerica, MA, USA), following manufacturer’s instructions (Bruker Daltonik, GmbH, Bremen, Germany), and considered a species-level identification when the ID score was >1.80 using the MALDI Biotyper version 3.0 software. Correctly identified isolates were stored in cryovials containing 1 mL of Tryptic Soy Broth with 20% glycerol at −80 °C until further analysis.

### 2.3. Antibiotics Susceptibility Testing (AST)

All isolates were tested for antibiotic susceptibility using the agar disc diffusion method (Kirby–Bauer), following EUCAST guidelines [13]. Enterobacterales and Lactobacillales were tested against 7 different antimicrobials belonging to 6 different antimicrobials classes; Bacillales were tested against 10 antimicrobials from 9 classes. For *Staphylococcus* spp., cefoxitin was also tested to screen for methicillin resistance [14]. All the discs were purchased from Oxoid (Oxoid S.p.A., Milan, Italy). Antimicrobials tested are listed in Table 1.

For the interpretation of inhibition zone diameters, each isolate was classified into wild type (WT) or non-wild type (NWT) using the standardized epidemiological cut-off values (ECOFFs) from EUCAST as reference values. This evaluation was carried out for the antimicrobials with published ECOFF value for the investigated bacterial genera. Isolates were considered WT when susceptible to all tested antimicrobial, and NWT when indicating the presence of phenotypically detectable acquired resistance mechanisms to at least one tested antimicrobial. Moreover, isolates showing no inhibition zone (diameter < 6 mm, indicating bacterial growth extending close to the edge of the disk without a discernible inhibition zone [15]) for the tested antimicrobials were classified as NWT, given clear evidence of phenotypic resistance, even in absence of ECOFF values. For *Staphylococcus* spp. isolates, interpretation of cefoxitin inhibition diameters followed the 2023 EUCAST clinical breakpoints [16] and isolates were subsequently classified as cefoxitin susceptible (S) or resistant (R) [17]. Intrinsic resistances for each bacterial species were excluded from AST evaluation, in accordance with EUCAST (2023) guidelines on expected resistance phenotypes [18].

### 2.4. Molecular Detection of Methicillin-Resistant Staphylococci

*Staphylococcus* spp. isolates exhibiting phenotypically cefoxitin resistance were subjected to molecular analysis by a multiplex PCR to detect methicillin-resistance genetic determinants. Bacterial DNA was extracted using a standard boiling method. The isolates were screened for the presence of *mecA* and *mecC* genes, according to the protocol described by Stegger et al. (2012) [19]. *Staphylococcus aureus* ATCC 43300 (*mecA*-positive) and *Staphylococcus aureus* BAA-2313 (*mecC*-positive) were used as positive control, while a *Staphylococcus* spp. strain, previously confirmed to be negative for both investigated genes, and sterile water, were used as negative controls. Primers and expected amplicon sizes for the investigated genes are detailed in Table 2.

### 2.5. Quality Control

To ensure the reliability of incubation conditions, culture media performance, as well as the quality and accuracy of AST procedures, American Type Culture Collection (ATCC) standard reference *E. coli* ATCC 25922, *Staphylococcus aureus* ATCC 25923 and *Staphylococcus aureus* ATCC 43300 strains were used to verify test performances.

### 2.6. Statistical Analysis

Descriptive statistical analysis was performed to describe the occurrence of antimicrobial resistance (AMR), including overall percentages of NWT distribution and resistance to single antimicrobials, within the sampled population and their bacterial isolates. Specifically, swifts were grouped by age (adults at the arrival/adults at the end of breeding/juveniles) and sampling period (arrival/end of breeding period), and this classification was also applied to their corresponding bacterial isolates. Associations between the outcome variables (single antimicrobial tested and NWT proportions) and the included variables (birds’ age and sampling period) were assessed using Fisher’s test or Pearson’s or Yates chi-squared (χ^2^) test, depending on the number of considered events. Differences in AMR prevalence between bacterial orders were also statistically evaluated using Fisher’s exact test. A *p*-value of ≤0.05 was considered statistically significant. Statistical analyses were conducted with EasyMedStat^®^ (version 3.29).

## 3. Results

### 3.1. Population Sampling and Data Collection

A total of 47 swifts were sampled across the four sampling sessions at the wildlife rehabilitation center. Of these, 20 were identified as common swifts (*Apus apus*), 2 as pallid swifts (*Apus pallidus*) and 1 as alpine swift (*Tachymarptis melba*), while species identification could not be determined for the remaining 24 birds. Sex determination revealed 15 female, 29 male and 3 individuals of undetermined sex. Age classification showed a total of 13 adult individuals and 34 juveniles (including 3 fledging and 31 nestlings).

A total of 10 adult swifts were sampled shortly after their arrival in Italy: 6 sampled in July 2023 and 4 in June 2024. Thirty-seven swifts were sampled at the end of the breeding season, before leaving for autumn migration. Specifically, 3 adult swifts were sampled in September 2023 and 34 juvenile swifts sampled as follows: 11 in July 2023 (1 fledgling and 10 nestlings), 5 nestlings in September 2023, 6 nestlings in June 2024 and 12 in August 2024 (2 fledglings and 10 nestlings).

### 3.2. Bacterial Isolation and Identification

**Figure 1:**
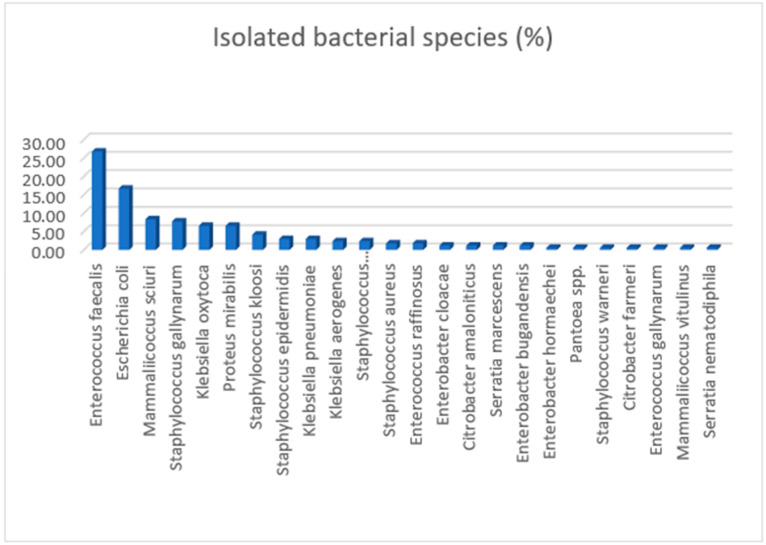
Percentage distribution of bacterial species isolated from all sampled swifts in the present study.

From the 47 sampled birds, a total of 168 bacterial isolates were obtained. These comprised 71/168 (42.26%, 95% CI 34.69 to 50.11) Enterobacterales, 49/168 (29.17%) Lactobacillales and 48/168 (28.57%) Bacillales. A total of 10 different bacterial genera and 24 distinct bacterial species were identified (Figure 1). The most frequently identified species were *Enterococcus faecalis* (n = 45; 26.79%), *Escherichia coli* (n = 28; 16.67%), *Mammaliicoccus sciuri* (n = 14; 10.12%), and *Staphylococcus gallinarum* (n = 13; 7.74%). The distribution of isolates by bacterial order among swifts at the two sampling points is presented in Table 3.

### 3.3. Antibiotics Susceptibility Testing (AST)

Of the 168 bacterial isolates recovered from all sampled swifts, 117 (69.64%, 95% CI 62.09 to 76.49) were classified as WT, whereas 51 (30.36%, 95% CI 23.51% to 37.91%) were NWT. The prevalence of NWT isolates emerged particularly among Bacillales isolates (28/48; 58.33%, 95% CI 43.21 to 72.39), followed by Lactobacillales (9/49; 18.37%, 95% CI 8.76 to 32.02) and Enterobacterales (13/71; 18.31%, 95% CI 10.13 to 29.27). Non-wild type isolates were detected towards all antimicrobial tested drugs, with highest percentages towards clarithromycin (33.33%), erythromycin (31.50%), clindamycin (21.88%) and tetracycline (14.29%). Additionally, AST revealed the presence of NWT phenotypes also to critically important antimicrobial classes, such as ertapenem 6/119 (5.04%, 95% CI 1.87 to 10.65). Moreover, 25/45 (55.6%, 95% CI 40 to 70.36) of isolates were classified as resistant to cefoxitin.

**Figure 2:**
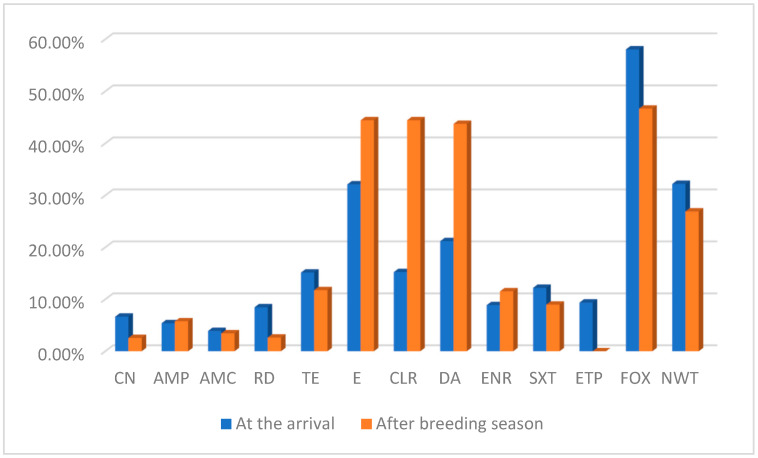
Non-wild type proportions among bacterial isolates from sampled swifts. This figure summarizes the percentage of NWT isolates for each tested antimicrobial drug, except cefoxitin, for which the percentage of resistant isolates is reported, highlighting differences in resistance prevalence between the two sampling periods. The last two bars show the total percentage of NWT isolates across all antimicrobials at the two sampling periods. Denominators (number of tested isolates) varied among antimicrobials and are reported in Table 4.

A summary of NWT proportion for each tested antimicrobial drug from swifts at both sampling points is provided in Figure 2 and Table 4.

#### Comparison of NWT Proportions Across Sampling Periods and Age Classes

A comparative analysis of NWT profiles associated with both sampling periods (at arrival/after breeding season) and the age class (adults/juveniles) revealed notable differences, summarized in Table 5.

When all isolates were considered, those from swifts sampled upon their arrival exhibited NWT proportion of 29/90 (32.22%; 95% CI 22.75 to 42.90), while those from swifts sampled before leaving for autumn migration showed NWT of 21/78 (26.92%, 95% CI 17.50 to 38.16) (Figure 2). At the arrival, higher NWT percentages were observed for tetracycline (15.56%), rifampicin (8.47%) and ertapenem (9.36%), whereas 58.1% of isolates were resistant to cefoxitin. Conversely, isolates obtained from swifts before migration showed a higher number of NWT isolates to erythromycin and clarithromycin (44.44%), clindamycin (43.75%) and enrofloxacin (11.54%).

When considering only adult swift isolates, from those sampled at their arrival 10/30 (33.33%, 95% CI 17.29 to 52.81) showed NWT phenotypes, with higher percentages to rifampicin (5.26%), compared with adults sampled before the autumn migration, with 5/13 isolates showing similar NWT prevalence (38.46%, 95% CI 13.86 to 68.42), but with a higher percentage towards enrofloxacin (23.08%).

Age-related comparisons highlighted similar overall NWT proportions between adults and juveniles. Nonetheless, juvenile isolates exhibited higher NWT percentages to several antibiotics, particularly rifampicin (5.63%), ertapenem (8%), and 54.3% were resistant to cefoxitin, mirroring the pattern observed in adults sampled at their arrival. Likewise, percentages of NWT isolates to clarithromycin (34.21%) and erythromycin (31.60%) resemble that of adults sampled before migration. Moreover, low percentages for ampicillin (4.85%) and amoxicillin–clavulanic acid (3.45%) were observed only in juveniles.

### 3.4. Molecular Detection of Methicillin-Resistant Staphylococci

Among the 48 *Staphylococcus* spp. isolates, 25 (52.08%, 95% CI 37.19 to 66.71) were phenotypically resistant to cefoxitin. From them, 22 (88%, 95% CI 68.78 to 97.45) carried the *mecA* gene, as confirmed by multiplex PCR indicating the presence of methicillin-resistant staphylococci (MRS), whereas the *mecC* gene was not detected in any of them. Notably, most *mecA* positive isolates were more frequently from swifts at their arrival (60%), compared to those sampled before migration (16%).

### 3.5. Statistical Analysis

Univariable statistical analysis revealed a significant association between the prevalence of NWT isolates and bacterial order. Bacillales isolates showed a significantly higher proportion of NWT isolates compared with Enterobacterales and Lactobacillales, both from adults and juveniles (*p* < 0.0001) and at both sampling points (*p* < 0.0001).

## 4. Discussion

In the present study, we investigated the presence of NWT bacteria, indicative of acquired phenotypical resistance mechanisms, within selected bacterial orders (Enterobacterales, Bacillales and Lactobacillales) of the commensal microbiota of swifts (*Apus* spp.), a long-distance migratory species covering migration areas across Africa and Europe. Several members of the bacterial orders investigated in this study can act as opportunistic pathogens, potentially causing infections in wild birds. For instance, Enterobacterales are known to lead to colibacillosis [8], while certain Bacillales and Lactobacillales have occasionally been associated with respiratory or gastrointestinal diseases in birds [9,10]. Therefore, detecting AMR in these commensal bacteria is relevant not only for environmental surveillance but also for understanding the potential health risks in wild bird populations. The main aim was to evaluate their role as bioindicators of environmental AMR. Despite growing attention to the role of wild migratory birds as bioindicators of environmental AMR contamination within the One Health framework, studies specifically exploring the role of swifts as valuable bioindicators of the spread of AMR in natural ecosystems are still lacking. Swifts show several ecological traits that already make them sensitive indicators: they are long-lived species (up to 12 years), breeding in colonies in urban areas, and feeding exclusively on aerial insects and airborne particles [20]. Previous studies have already demonstrated their role in biomonitoring environmental quality, including the accumulation of persistent organic contaminants and airborne particulates [21]. From our findings, the commensal microbiota of sampled swifts, within the bacterial orders targeted in this study, resulted predominantly composed of *Enterococcus faecalis* and *Escherichia coli*, the most frequently recovered bacteria species. This is consistent with previous studies reporting these taxa as ubiquitous bacteria inhabiting various environmental habitats and commonly found as commensals in mammals, birds, reptiles and invertebrates [22,23]. However, our result is mainly attributable to the use of selective media: MacConkey agar for selective isolation of Enterobacterales, which contributed to the predominant isolation of *E. coli*, a dominant commensal in the intestinal microbiota of wild birds; and Mannitol Salt Agar which favors the growth of Bacillales and Lactobacillales, like *Enterococcus* spp. and staphylococci. Among these, coagulase-negative staphylococci (CoNS), particularly *Mammaliicoccus sciuri* and *Staphylococcus gallinarum*, were the most frequently recovered, aligning with previous observations reporting that birds are more frequently colonized by CoNS than mammals [24]. *M. sciuri* (formerly known as *Staphylococcus sciuri*) has been described as one of the most prevalent CoNS in avian species and widely distributed in nature [25], while *S. gallinarum*, although less frequently reported, has also been isolated from various animal species, including birds [24].

Overall, 30.36% of isolates revealed a non-wild type (NWT) phenotype, indicative of the occurrence of phenotypically detectable acquired resistance. The levels of antimicrobial resistance reported in wild birds vary widely across studies, reflecting heterogeneous available data. Similar prevalence has been documented, such as in a study from Spain reporting an AMR rate of 47.4% in white storks and seagulls [26]. Higher AMR prevalence has also been described in other migratory species, such as in the study by Akhil Prakash et al. (2022), which reported AMR rates of 94% in the commensal microbiota of Arctic terns [27]. However, in our study, the prevalence of an acquired resistance may have been underestimated. This may be partly due to the interpretative criteria applied, which were based primarily on ECOFFs. For several bacterial species and antimicrobials, tested specific ECOFF values are currently unavailable, leading to the inclusion of no inhibition zone isolates within NWT category. This adoption should be considered for future environmental and non-clinical bacterial isolates to significantly enhance the AMR assessment and the detection of acquired resistance circulation in the context of environmental monitoring.

Specifically, in our study, a higher NWT prevalence was observed among Bacillales (58.33%), compared to Lactobacillales (18.37%) and Enterobacterales (18.31%). This finding was supported by statistical analysis, which confirmed a significant association between NWT proportion and Bacillales isolates from swifts sampled at both two sampling periods and both adults and juveniles (*p* < 0.0001). Notably, it has been widely reported that *Staphylococcus* spp. possesses a broad host range and strong environmental adaptability, allowing them to persist long-term in diverse habitats where antibiotic pressure may drive the selection and spread of resistant strains, potentially making them indicators of environmental contamination [7,24].

Among all bacterial isolates, the higher percentages of NWT phenotypes were observed for clarithromycin (33.33%), erythromycin (31.50%), clindamycin (21.90%), and tetracycline (14.29%), whereas 55.6% isolates were resistant to cefoxitin. Moreover, particularly concerning was the detection of NWT isolates even to last-resort antimicrobials, such as ertapenem (5.04%), and the presence of critical resistance determinants like *mecA* gene (76%). Carbapenem resistance represents a global critical issue, as these antibiotics represent one of the last therapeutic options against multidrug-resistant bacteria. Although typically associated with Enterobacterales [28], in our study, carbapenem NWT phenotypes emerged largely among *Staphylococcus* isolates, showing no inhibition halos, possibly reflecting selective pressures and the circulation of resistant strains even within Gram-positive bacteria. Indeed, carbapenem resistance has been reported among multidrug-resistant *Staphylococcus* spp., particularly methicillin-resistant staphylococci [29], already characterized by multiple resistance, but, without genetic confirmation of carbapenemase production in our isolates, the clinical relevance of these findings remains uncertain. The high prevalence of cefoxitin resistance, combined with the genotypic detection of the *mecA* gene, confirmed the presence of methicillin-resistant staphylococci (MRS) among our isolates, raising questions about the possible acquisition of such critical resistance genes. Similar research by Abdullahi et al. (2023) reported methicillin resistance in 100% of *S. aureus* from migratory white storks, attributing it to nest exposure to human residues, considered a risk factor for AMR acquisition [30]. This finding supports a possible explanation to our result, considering the swifts’ habit of nesting in strictly urban environments. However, the detection of carbapenem resistance and MRS, both representing major concern in human and veterinary medicine, underscores the need for continued environmental surveillance of AMR and a deeper investigation of the role of migratory birds as potential bioindicators or reservoirs of critical resistance determinants at human–wildlife–environment interface.

Our findings primarily aim to highlight the prevalence of AMR within the targeted commensal bacterial orders of swifts. Beyond overall AMR prevalence, comparative insights emerged regarding acquired antimicrobial resistance patterns among isolates from grouped swifts. These included isolates from swifts sampled shortly after the arrival in Italy, swifts that had possibly spent the breeding season in Italy, and juveniles’ swifts born in Italy. Some subgroups included small sample sizes; therefore, comparisons and observed differences should be interpreted as exploratory rather than definitive. Swifts, as long-distance migratory birds spending most of their active time flying and moving between different areas of the world as part of their annual life cycle [31], may be able to provide significant insights on AMR contamination even across distinct environmental contexts. Understanding the swift’s migratory cycle is fundamental to evaluating their exposure to different ecosystems. So far, migration routes and exact wintering areas of swifts remain poorly defined due to limited ringing recoveries, but it has been recently reported that during winter, the swift migrates to sub-Saharan Africa stopover sites, spanning regions such as the Sahel, the Congo Basin, and parts of East and Southern Africa, conducting their aerial lifestyle and continuous mobility during the non-breeding season [32]. In Europe, and in Italy, the swift’s typical arrival is in early spring for the breeding period, departing in late summer [32]. Their unique ecological cycle exposes swifts to different environmental contexts, even across continents, making them interesting candidates as bioindicators of environmental AMR.

Isolates from swifts sampled shortly after their arrival revealed a NWT proportion of 32.22%, compared to 26.92% in isolates from swifts sampled before the autumn migration. Moreover, when considering only adults swifts, the NWT percentage was 34.88%, compared to juveniles (28.8%). These results may be indicative of a potential exposure to antimicrobial resistant bacteria during migration routes, although direct evidence is lacking. Swifts’ wintering sites, usually across several African regions, could be characterized by widespread and often unregulated use of antimicrobials in human and veterinary medicine, combined with a lack of surveillance AMR programs, which might strongly create a substantial selective pressure for resistant bacterial strains [33]. Nevertheless, the presence of NWT isolates in juveniles that had never migrated may suggest rapid acquisition from the environment or from parents’ transmission.

Isolates from swifts at the arrival showed elevated resistance rate against cefoxitin (58.06%) and NWT phenotypes to ertapenem (9.38%), consistent with the circulation of MRS detected in this study, as previously described. High NWT percentage to tetracycline (15.56%) was also observed, possibly reflecting its extensive use in veterinary contexts. Tetracycline is considered the main antimicrobial drug used in poultry farming, particularly in African areas [34], where poultry is considered a dominant livestock sector [35]. Finally, NWT isolates toward rifampicin (8.47%) were also observed, a widely used antibiotic drug for the treatment of tuberculosis [36]. Interestingly, in a previous study from our research group, we highlighted a correlation between rifampicin phenotypical resistance and isolates from migratory wild birds [7], suggesting a possible trend in migratory birds’ species and epidemiological significance of rifampicin acquired resistance from African areas, but further studies are needed to clarify this association.

On the other hand, isolates from swifts sampled before autumn migration, after spending the breeding season in Europe, possibly in Italy, showed a marked increase in NWT percentages toward macrolides, specifically clarithromycin (44.44%) and erythromycin (44.44%), clindamycin (43.75%) and enrofloxacin (11.58%). The results that emerged may suggest the possible acquisition of locally originated acquired resistance during the breeding season. Swifts predominantly nest in urban areas where contact with contaminated surface waters, urban waste, or their diet based on insects from polluted environments may enhance exposure to resistant strains. According to the Italian National Antibiotic Use Report from 2023, macrolides are among the most frequently used in the Italian population [37]. It is important to note that macrolides belong to the Watch group of antibiotics in the AWaRe classification introduced by the World Health Organization in 2022 [38]. Hence, these antibiotics are broad-spectrum molecules, recommended only as first-choice treatment towards pathogens more likely to be resistant to narrow-spectrum options. Furthermore, macrolides environmental persistence, especially for clarithromycin, has been well documented [39], emphasizing the importance of paying attention to this antimicrobial class both for its clinical relevance and impact at environmental level. Clindamycin is an antibiotic widely used in birds, especially for the treatment of *Staphylococcus* spp. infections, and resistance is frequent in staphylococci isolated from wildlife [40]. For what concerns fluoroquinolones, they have also been frequently used in veterinary medicine [41], and according to European Medicines Agency (EMA), Italy still records high antimicrobial consumption rates in veterinary medicine across Europe [42]. A possible explanation for the higher prevalence of NWT isolates across sampled swifts after the breeding season may be linked to the conditions of the wildlife rehabilitation center where sampling occurred. In such settings, the aggregation between individuals, possible environmental contamination, and occasional exposure to antimicrobial treatments could facilitate the selection and circulation of resistant bacteria [43]. Specifically, enrofloxacin is one of the most administrated antibiotics in birds [44], which implies a plausible direct or indirect exposure in individuals in rehabilitation center.

Age-stratified analysis, comparing adult and juvenile swift isolates, revealed further details. Indeed, juveniles isolates showed higher NWT proportion to clarithromycin (34.21%), erythromycin (31.60%) and enrofloxacin (9.91%), similar to isolates from swifts sampled before the autumn migration, strengthening the hypothesis of a local acquisition during the breeding season, whereas NWT percentage to rifampicin (5.63%) was comparable between juveniles and isolates from swifts sampled at their arrival, supporting the possible acquisition of certain resistance during the wintering grounds. These findings may be related to an early-life exposure to resistant bacteria, possibly acquired through feeding behaviors from parents; a hypothesis supported by the literature reporting that birds’ gut microbiota is largely shaped by maternal transfer diet [45]. Moreover, an immature immune system of nestlings, which may facilitate the colonization and the persistence of resistant strains, but also the nest microenvironment, characterized by high bacterial density, could represent a hotspot for the resistance genes exchange [46]. Considering the swift’s ecological behaviors, its tendency to nest in urban settings make it a highly exposed species to anthropogenic contaminants, including AMR bacteria and resistance determinants. The low NWT phenotypes to ampicillin (4.85%) and amoxicillin–clavulanic acid (3.45%) observed exclusively in juveniles may further highlight potential antimicrobial pressure in urban setting or at the recovery center, since these antimicrobials are used as therapeutic agents in both human and veterinary medicine [47]. Nonetheless, specific contributions of all these potential transmission routes and the long-term consequences of early-life microbial exposure in birds remain to be clarified.

Finally, the detection of antimicrobial-resistant bacteria in migratory birds also carries important ecological and conservation implications. Environmental antimicrobial resistance can be considered as a cumulative outcome of anthropogenic impacts on natural ecosystems, a problem that not only involves the human community, but that also affects wildlife [4]. Migratory species such as swifts, characterized by aerial lifestyle, an exposure across multiple habitats and geographical regions during their life cycle, and nesting in urban and peri-urban areas, may increase their potential direct and indirect contact to environmental AMR. Consequently, the presence of resistant bacteria in their commensal microbiota may have implications for their health, microbiome stability and susceptibility to opportunistic infections [10], not only for the species itself but also for other avian hosts, particularly in shared settings such as wildlife rehabilitation centers. In this context, wild birds, and swifts in particular, may provide relevant information not only for environmental AMR surveillance, but also for wildlife management and conservation strategies aimed at preserving ecosystem and wildlife health.

Despite the relevance of these findings in advancing environmental monitoring of AMR through the evaluation of NWT profiles from the investigated commensal bacterial microbiota of swifts, they should be interpreted with caution due to certain limitations. First, NWT profiles emerged from swifts sampled at their arrival have been related to a possible exposure during wintering periods in African regions; however, the possibility of early AMR acquisition within the rehabilitation center cannot be excluded. Similarly, NWT profiles from swifts sampled at the end of the breeding period could have acquired resistance phenotypes locally or be chronic AMR carriers. This highlights an intrinsic bias introduced by sampling individuals recovered, even temporarily, in a captive environment where exposure to human- or environmental-related bacterial communities may occur. Additionally, individuals were not sampled longitudinally across both time points, limiting the ability to track within-individual AMR acquisition. Another limitation is a relatively small sample size and the consequent underrepresentation of adult swifts at the arrival in Italy. Furthermore, genomic analyses such as Whole Genome Sequencing (WGS) could provide insights into the genetic basis of AMR determinants. Therefore, future research should aim for larger and more balanced samples, longitudinal monitoring of the same individuals, sampling in natural habitats rather than rehabilitation centers, and inclusion of advanced genomic analyses, to allow a more accurate evaluation of the potential role of swifts as bioindicators of environmental AMR dissemination.

## 5. Conclusions

This study provides novel insights supporting the unexplored role of the swift (*Apus* spp.), a long-distance migratory bird moving from sub-Saharan Africa to Europe, as bioindicator for environmental AMR monitoring. Our findings demonstrated the presence of non-wild type (NWT) bacteria within the commensal microbiota of swifts, specifically among selected bacterial orders (Enterobacterales, Bacillales and Lactobacillales), highlighting the presence of acquired phenotypical resistance. Comparative analysis revealed partially overlapping NWT prevalences, but also the emerging of some differences across sampling periods and age classes, suggesting that swifts may be exposed to resistant bacteria under different environmental contexts throughout their migratory life cycle, both in wintering grounds as well as in breeding areas. High percentages of NWT isolates observed in swifts at the arrival may reflect the selective pressure encountered along their migratory routes (tetracycline, rifampicin), whereas those observed in swifts sampled after the breeding season (macrolides, clindamycin, enrofloxacin) may be associated with local selective pressures or those present within recovery centers. Moreover, the detection of methicillin-resistant staphylococci and NWT isolates to last-resort antimicrobials emphasize the circulation of resistant bacteria and ARGs across humans, wildlife and environment interface. In conclusion, from a One Health perspective, our findings suggest a promising role of swifts (*Apus* spp.) as bioindicators of AMR contamination and reinforce the added value of incorporating wildlife into effective AMR environmental surveillance strategies, contributing to a more comprehensive understanding of AMR dissemination across human, animal and environmental compartments.

## Figures and Tables

**Table 1:** List of tested antimicrobials grouped by antimicrobial class with corresponding code abbreviation/disk concentration (µg), bacterial order tested, availability of ECOFFs value for specific bacterial species, and EUCAST clinical zone diameter breakpoints applied.

Antimicrobial Class	Antimicrobial	Code (μg)	Bacterial Order Tested	ECOFFs (NWT < mm)	EUCAST Clinical Zone Diameter Breakpoints (R < mm)
Aminoglycosides	Gentamicin	CN (10)	Bacillales,	*S. aureus* (18); *S. epidermidis* (20)	
			Enterobacterales	*E. coli*, *P. mirabilis*, *Serratia marcescens* (16); *K. oxytoca*, *C. freundii*, *E. cloacae* (15); *K. pneumoniae*, *K. aerogenes* (14);	
Penicillins + beta-lactamase inhibitors	Ampicillin	AMP (10)	Enterobacterales,	*E. coli* (13); *P. mirabilis* (19)	
			Lactobacillales	*E. faecalis* (12)	
	Amoxicillin + clavulanic acid	AMC (30)	Enterobacterales	*E. coli* (14); *K. pneumoniae* (22); *P. mirabilis* (22)	
			Lactobacillales	N. A.	
Cephalosporins	Cefoxitin	FOX (30)	Bacillales	*-*	*S. aureus*, CoNs (22); *S. epidermidis* (27)
Rifamycins	Rifampicin	RD (5)	Bacillales	*S. aureus* (24); *S. epidermidis* (30)	
			Lactobacillales	N. A.	
Lincosamides	Clindamycin	DA (2)	Bacillales	*S. aureus*, *S. epidermidis* (21)	
Tetracyclines	Tetracycline	TE (30)	Bacillales,	*S. aureus* (20)	
			Enterobacterales	*E. coli* (21)	
			Lactobacillales	N. A.	
Macrolides	Erythromycin	E (15)	Bacillales	*S. aureus* (22)	
	Clarithromycin	CLR (15)	Bacillales	N. A.	
Fluoroquinolones	Enrofloxacin	ENR (5)	Bacillales,	N. A.	
			Lactobacillales,	N. A.	
			Enterobacterales	N. A.	
Sulfonamides + dihydrofolate reductase inhibitors	Trimethoprim-sulfamethoxazole	SXT (25)	Bacillales,	*S. aureus* (23)	
			Lactobacillales,	*E. faecalis* (26)	
			Enterobacterales	*E. coli* (22); *K. oxytoca* (21); *K. pneumoniae* (18); *E. cloacae*, *P. mirabilis* (20)	
Carbapenems	Ertapenem	ETP (10)	Enterobacterales,	*E. coli* (24); *K. pneumoniae* (22)	
			Bacillales	N. A.	

**Table 2:** Primers sequences and expected product size used for the detection of methicillin-resistance genes in the present study.

Gene	Sequences (5′–3′)	Product Size (bp)	References
*mecA*	Fw: TCCAGATTACAACTTCACCAGG	162	[19]
	Rev: CCACTTCATATCTTGTAACG		
*mecC*	Fw: AAAAAAAGGCTTAGAACGCCTC	138	[19]
	Rev: GAAGATCTTTTCCGTTTTCAGC		

**Table 3:** Distribution of bacterial isolates recovered from grouped swifts, according to bacterial order. Percentages for each category are calculated relative to the total number of isolates per group.

Grouped Swifts	Tot Isolates (n)	Enterobacterales (n); (%)	Bacillales (n); (%)	Lactobacillales (n); (%)
TOTAL	168	71; (42.26%)	48; (28.57%)	49; (29.17%)
AT THE ARRIVAL	90	31; (34.44%)	33; (36.67%)	26; (28.89%)
AFTER BREEDING SEASON	78	40; (51.28%)	15; (19.23%)	23; (29.49%)

**Table 4:** NWT percentages among isolates at two sampling points. Data are reported as percentages (n) for the total of isolates considered NWT in the present study, NWT based on available ECOFF values, and NWT based on absence of inhibition zone (zero halo) for each antibiotic tested. For cefoxitin (FOX) only, R (resistant) is reported instead of NWT.

	At the Arrival			After Breeding Season		
	NWT/R/Zero Halo	NWT (ECOFFs)	Zero Halo	NWT/R/Zero Halo	NWT (ECOFFs)	Zero Halo
CN	6.66% (6/90)	16.21% (6/37)	0% (0/53)	2.56% (2/78)	4.87% (2/41)	0% (0/37)
AMP	5.40% (2/37)	6.25% (2/32)	0% (0/5)	5.76% (3/52)	5.76% (3/52)	0% (0/26)
AMC	3.92% (2/51)	8.33% (1/12)	2.56% (1/39)	3.44% (2/58)	6.25% (2/32)	0% (0/26)
RD	8.47% (5/59)	0% (0/7)	9.61% (5/52)	2.63% (1/38)	0% (0/1)	0% (0/37)
TE	15.55% (14/90)	45.45% (5/11)	11.39% (9/79)	11.76% (8/68)	0% (0/20)	16.66% (8/48)
E	32.14% (9/28)	50% (1/2)	30.76% (8/26)	44.44% (8/18)	100% (1/1)	41.17% (7/17)
CLR	15.25% (9/59)	−	15.25% (9/59)	44.44% (8/18)	−	44.44% (8/18)
DA	21.21% (7/33)	28.57 (2/7)	19.23% (5/26)	43.75% (7/16)	0% (0/1)	46.66% (7/15)
ENR	8.88% (8/90)	−	8.88% (8/90)	11.53% (9/78)	−	11.53% (9/78)
SXT	12.22% (11/90)	8.33% (4/48)	16.66% (7/42)	8.97% (7/78)	11.66% (7/60)	0% (0/18)
ETP	9.37% (6/64)	9.09% (1/11)	9.43% (5/53)	0% (0/55)	0% (0/22)	0% (0/33)
FOX	58.06% (18/31)	−	−	46.66% (7/15)	−	−

**Table 5:** NWT percentages, and in brackets the number of NWT isolates, for each antimicrobial tested in the total of bacteria isolates from swifts grouped according sampling period and age. For cefoxitin, the percentage of resistant isolates is reported. The percentage value of the total NWT isolates for each category is given in the last row.

	Adults at the Arrival	Adults After Breeding Season	Juveniles
CN	3.33% (1/30)	0% (0/13)	5.60% (7/125)
AMP	0% (0/24)	0% (0/10)	4.85% (5/103)
AMC	0% (0/29)	0% (0/13)	3.45% (4/116)
RD	5.26% (1/19)	0% (0/7)	5.63% (4/71)
TE	16.67% (5/30)	15.38% (2/13)	13.51% (15/111)
E	22.22% (2/9)	20% (2/10)	31.60% (12.38)
CLR	22.22% (2/9)	20% (2/10)	34.21% (13/38)
DA	22.22% (2/9)	20% (2/10)	26.31% (10/38)
ENR	10% (3/30)	23.08% (3/13)	9.91% (11/111)
SXT	3.33% (1/30)	15.38% (2/13)	9.91% (11/111)
ETP	0% (0/20)	0% (0/10)	8% (6/75)
FOX	44.44% (4/9)	50% (2/4)	54.29% (19/35)
**NWT**	**33.33** **% (10/** **30** **)**	**38.46% (5/13** **)**	**28.8% (36/125)**

## Data Availability

The raw data generated and analyzed during the current study are not publicly available but can be made available by the authors on request.

## Abbreviations

The following abbreviations are used in this manuscript:

AMR	Antimicrobial resistance
MALDI-TOF MS	Matrix-assisted laser desorption–ionization time- of-flight mass spectrometry
WT	Wild type
NWT	Non-wild type
CN	Gentamicin
AMP	Ampicillin
AMC	Amoxicillin + clavulanic acid
FOX	Cefoxitin
RD	Rifampicin
DA	Clindamycin
TE	Tetracycline
E	Erythromicyn
CLR	Clarithromycin
ENR	Enrofloxacin
SXT	Trimethoprim-sulfamethoxazole
ETP	Ertapenem
PCR	Polymerase chain reaction
AST	Antimicrobial susceptibility test
CoNS	Coagulase-negative staphylococci
MRS	Methicillin-resistant staphylococci
ARGs	Antimicrobial resistance genes
